# Using Parasitic Load to Measure the Effect of Anthropogenic Disturbance on Vervet Monkeys

**DOI:** 10.1007/s10393-018-1349-y

**Published:** 2018-08-08

**Authors:** Harriet R. Thatcher, Colleen T. Downs, Nicola F. Koyama

**Affiliations:** 1 School of Natural Sciences and Psychology, Liverpool John Moores University, Byrom St, Liverpool, L3 3AF UK; 2 School of Life Sciences, University of KwaZulu-Natal, Pietermaritzburg Campus, KwaZulu-Natal, South Africa

**Keywords:** Zoonosis, Transmission, Human–wildlife, Land-use gradient, Management

## Abstract

Vervet monkeys, *Chlorocebus pygerythrus*, thrive in urban areas of KwaZulu-Natal, South Africa, and present a suitable model to assess parasitic load as a measure of anthropogenic disturbance, such as urbanization. We collected vervet monkey faecal samples from four study sites representing a gradient of land use and urbanization. We assessed faecal parasites using the faecal flotation method calculating eggs per gram and parasite richness. Overall, the more urban vervet monkey populations had a significantly higher parasite richness and abundance. Our study shows the applicability of using parasite load to measure the effect of urbanization on wildlife.

Dramatic increases in human populations have resulted in drastic changes to the function and biodiversity of the natural ecosystem (Sauvajot 1998; Bonier et al. 2006). Though effects are species specific, certain wildlife species have been able to adapt to ecological changes and thrive in these conditions (McLennan et al. 2017). However, the stresses of an expanding anthropogenic environment can have negative consequences for wildlife such as poor body condition and increased parasite load (Borg et al. 2015; Soto-Calderón et al. 2016). Understanding the effects of anthropogenic disturbance, such as urbanization, on host–parasite relationships and zoonotic transmission has implications not only for the health of humans and their livestock, but also for wildlife conservation and biodiversity (Soulsbury and White 2015; Humle and Hill 2016; Cable et al. 2017).

Urbanization varies dramatically from large cities to small settlements, and therefore, the effects are difficult to quantitatively measure (Niemelä and Kotze 2009; Bennett and Gratton 2012; Mackenstedt et al. 2015). Urbanization creates unique habitats through a process of increasing human populations and anthropogenic structures (Werner 2011). With the continuing encroachment of anthropogenic pressures, most wildlife studies include some level of human disturbance in their data (e.g. McLennan et al. 2017). However, with the exception of Lane et al.’s (2011) study on *Macaca fascicularis*, few primate studies include highly human-populated urban areas. Furthermore, the majority of anthropogenic parasite studies focus on comparisons between only two study sites. However, the results of these studies show varying effects of increasing urbanization on zoonotic transmission and parasite infection rates (Cable et al. 2017). Creating a quantitative measure for urban influences is complex, but currently a gradient scale from rural to urban is the most commonly acknowledged method (Bradley and Altizer 2006; Shochat et al. 2007; Bennett and Gratton 2012).

Vervet monkeys, *Chlorocebus pygerythrus,* are a common generalist primate that has become highly populated within urban areas of KwaZulu-Natal, South Africa (Patterson et al. 2016; 2017; 2018). They are, therefore, a suitable model to investigate anthropogenic influences on wildlife parasite load. Furthermore, they are frequent raiders of anthropogenic food sources increasing opportunities for zoonotic transmission (Eley 1989; Hahn et al. 2003; Hegglin et al. 2015). Research into anthropogenic influences on parasite load in vervet monkeys is limited to two studies (Gaetano et al. 2014; Valenta et al. 2017). Gaetano et al.’s (2014) research suggests that ecological factors are better predictors of parasitism concentrations than anthropogenic contact. However, the applicability of their findings is limited by both small sample size and relatively short time span. Valenta et al. (2017) reported a higher parasite richness in vervet monkeys in an anthropogenic disturbed habitat compared with historical data in less disturbed vervet monkey populations. However, they were unable to interpret their results clearly due to variation in sample sizes and methods across historical studies. Finally, the habitats assessed in both studies had low anthropogenic stress, highlighting a need to assess a wider range of anthropogenic influences on vervet monkeys.

We aimed to establish whether a relatively low-cost methodological approach to assessing parasite load could reflect an urbanization gradient. We used four sites throughout KwaZulu-Natal, South Africa. We chose study sites to reflect anthropogenic influences along a rural–periurban–urban gradient (Table 1), including the previously neglected category of a highly human-populated urban area. Vervet monkey troop size information was collected using standard point count protocol (Hutto et al. 1986). We obtained human populations and anthropogenic structure numbers from site officials and governmental records (STATS SA, 2017) (Table 1).

**Table 1:** Information on Vervet Monkey Faecal Parasite Samples Collected from Four Sites Representing a Rural–Periurban–Urban Gradient in KwaZulu-Natal, South Africa.

Site	Human density per km^2^	Anthropogenic structure per km^2^	Group size	Mean no. samples (± SD) per month	McKinney classification
Private reserve	2	2	20	49 (9 ± 0.7)	CDEB
			49	27 (4.5 ± 1.21)	
			16	31 (5 ± 0.3)	
Industrial land	48	4	22	28 (4 ± 0.3)	DDIC
Gated estate	2970	275	12	24 (4 ± 0.2)	HG_3_LC
			23	23 (4 ± 0.3)	
			27	27 (5 ± 0.3)	
			42	31 (5 ± 0.3)	
City centre	3100	352	28	22 (4 ± 0.2)	HKPO
			35	24 (4 ± 0.4)	

Sites have also been Classified Using McKinney’s Standardized Classification System (2015).

We collected vervet monkey faecal samples over 6 months from October 2016–March 2017. We collected 286 specimens immediately after defecation following standard sampling techniques, storing them in 70% ethanol (Gillespie 2006).

Vervet monkey samples were prepared for analyses using the faecal flotation method (Gillespie 2006). We pipetted the prepared sample from the centre of the tube into chambers of a McMaster slide. The slide was assessed using standard methods (Cringoli et al. 2004). We used an electron phase microscope to scan the slide using a Å ~ 10 objective lens and identified parasite eggs based on morphology. Digital photographs were taken of any vervet monkey parasites observed.

We converted raw data from the McMaster analysis to eggs per gram (Dunn and Keymer 1986) and compiled information on parasite richness per sample. We classed vervet monkeys as infected if their faecal sample had one or more parasite(s) and present the percentage of samples infected.

We analysed data using R v3.3.2 (R Project 2017). Data for both eggs per gram and parasite richness were not normally distributed (Shapiro–Wilks test, *p *≤ 0.001) (Ghasemi and Zahediasl 2012). We ran two generalized linear models, with eggs per gram and parasite richness as separate dependent variables. We tested eggs per gram with a Poisson distribution and log link suitable for frequency data and species richness with a gamma distribution and log link for non-normal data. For both models, to avoid collinearity, we combined human density per km^2^ and anthropogenic structure per km^2^ to create a fixed effect. We also included vervet monkey troop size as a fixed effect. Generalized linear models were specified using the lme4 package (Bates 2010). To test whether the fixed effects explained variation we used a likelihood ratio test (‘anova’ command set to ‘Chisq’) to compare the maximum model against the null model (Zuur et al. 2009). Furthermore, we bootstrapped our confidence intervals to account for uneven sampling within our data set (Davison and Hinkley 1997).

Overall, 58% of the 286 vervet monkey samples had some level of parasitic infection. Parasites identified were *Coccidia* sp., *Strongyloides* sp., *Tricuris* sp., *Ascaris* sp. and *Oesophagostomum* sp. Eggs per gram were significantly higher in vervet monkeys from more urbanized sites (Table 2, Fig. 1a). Increasing vervet monkey troop size also had a significant positive effect on eggs per gram (Table 2, Fig. 1b). Parasite richness was significantly higher in vervet monkeys inhabiting more urbanized habitats (Table 3, Fig. 2a). Increasing troop size also had a significant positive effect on vervet monkeys’ parasite richness (Table 3, Fig. 2b).

**Table 2:** Maximum Model Output from Likelihood Ratio Test on the Eggs per gram of Vervet Monkey Faecal Samples (*n* = 286) Collected Along a Rural–Periurban–Urban Gradient in KwaZulu-Natal, South Africa.

Dependent variables	Fixed effects	Estimate	Standard error	Bootstrapped confidence intervals		Likelihood ratio test	
				Lower	Upper	Deviance	*p* (chi)
Eggs per gram	Intercept	1.45	1.64				
	Anthropogenic value km^2^	2.84	4.51	0.02	0.01	35.83	≤ 0.001
	Troop size	6.34	1.93	0.12	0.21	11.17	0.001

**Fig. 1:**
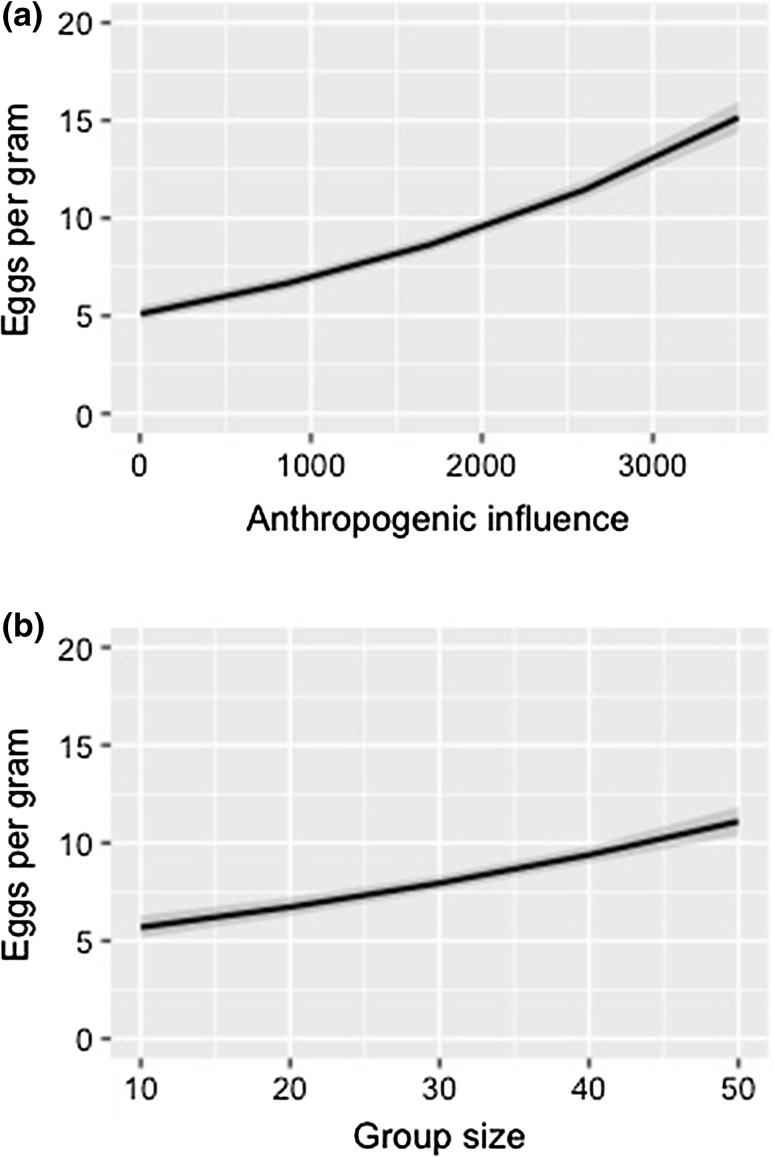
Eggs per gram obtained from vervet monkey faecal samples collected from four sites representing a gradient of urbanization in KwaZulu-Natal, South Africa, where **a** shows the positive significant effect of increased anthropogenic influence on eggs per gram of vervet monkeys (*p *≤ 0.001), and **b** shows the positive significant effect of troop size on eggs per gram of vervet monkeys (*p *= 0.001).

**Table 3:** Maximum Model Output from Likelihood Ratio Test on the Parasite Richness in Vervet Monkey Faecal Samples (*n* = 286) Collected Along a Rural–Periurban–Urban Gradient in KwaZulu-Natal, South Africa.

Dependent variable	Fixed effects	Estimate	Standard error	Bootstrapped confidence intervals		Likelihood ratio test	
				Lower	Upper	Deviance	*p* (chi)
Species richness	Intercept	− 8.87	2.39				
	Anthropogenic value km^2^	2.47	4.34	0.01	0.03	29.96	≤ 0.001
	Troop size	1.23	5.38	0.01	0.02	5.27	0.021

**Fig. 2:**
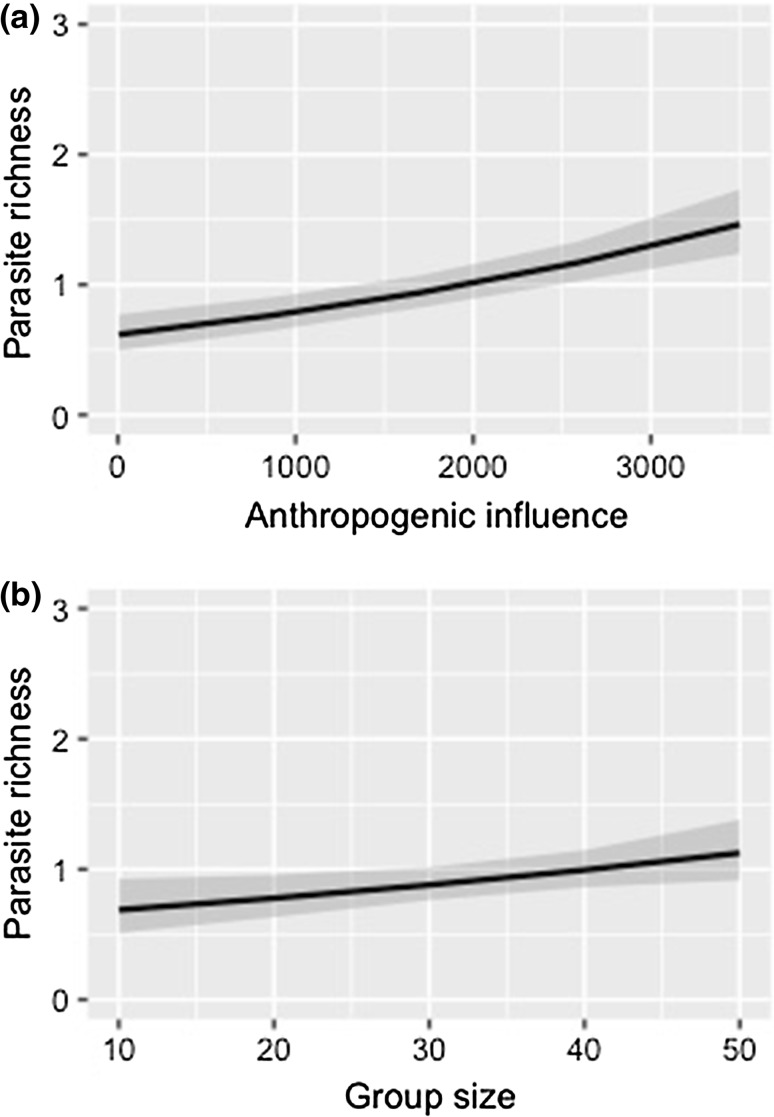
Parasite richness obtained from vervet monkey faecal samples collected from four sites representing a gradient of urbanization in KwaZulu-Natal, South Africa where **a** shows the positive significant effect of increased anthropogenic influence on parasite richness of vervet monkeys (*p *≤ 0.001), and **b** shows the positive significant effect of troop size on parasite richness of vervet monkeys (*p *= 0.021).

Parasite eggs per gram and species richness were significantly higher in vervet monkeys living in areas of higher human density and greater anthropogenic structure than in lower human density and anthropogenic structure, supporting previous studies (e.g. Valenta et al. 2017). As expected, vervet monkey troop size was a significant predictor across sites for both eggs per gram and parasite richness. Overall, our findings suggest that increased urbanization increases parasite load in vervet monkeys. Past results into the effects of anthropogenic disturbance on primate parasite load are mixed (Cable et al. 2017), although the majority of studies have compared only disturbed and undisturbed categories (Gillespie and Chapman 2008). Here, we analysed a gradient of urbanization, crucially including a highly human-populated urban area.

Anthropogenic pressures in dense urban environments, such as city centres, can have a negative impact both at an individual level (body condition: Eley 1989; Scheun et al. 2015) and at a group level (group stability: Sinha and Mukhopadhyay 2013). Supporting this, we found that parasite eggs per gram and richness were greater where anthropogenic pressures were higher. Increased anthropogenic influences result in increased contact with humans for wildlife that can facilitate disease transmission (Eley 1989; Hahn et al. 2003; Hegglin et al. 2015). A greater public awareness of the need to minimize food raiding opportunities for vervet monkeys could limit contact and reduce exposure to potential pathogens for both species. This is especially important considering the nature of foods raided. Both provisioned and raided foods generally contain a greater starch content which can contribute to increased parasite concentrations in host species (Weyher et al. 2006; Becker et al. 2015).

Although the vervet monkey parasite species found in our study were consistent with those found in other studies on urban primates, without genetic analysis we were unable to look at the direct transmission effects of these parasites, particularly as those we found are species specific. A greater understanding of zoonotic transmission would be a valuable asset, both from the perspective of human well-being and ecological biodiversity conservation (Díaz et al. 2006). Our findings highlight the suitability of the faecal flotation protocol as a relatively low-cost sampling method to monitor host–parasite responses to urbanization in species such as vervet monkeys. Such methodology could be included in urban management plans on a wider scale to assess the relationship between anthropogenic ecological change and wildlife health.

Our study is the first to provide baseline parasite data on vervet monkeys living in relatively highly urbanized areas. The wide range of sites used allowed us to conduct a controlled comparison of the effect of anthropogenic influences across a rural–periurban–urban gradient. Results highlight that increased urbanization is related to both increased eggs per gram and parasite richness in vervet monkeys. The study provides an important foundation for these successful urbanites. As urbanization increases, a greater understanding of urban exploiters’ adaptations to ecological changes is important.

The datasets generated during the current study are available from the corresponding author on reasonable request.
